# Hypotension and a positive fluid balance are associated with delirium in patients with shock

**DOI:** 10.1371/journal.pone.0200495

**Published:** 2018-08-07

**Authors:** Duc Nam Nguyen, Luc Huyghens, Jose Parra, Johan Schiettecatte, Johan Smitz, Jean-Louis Vincent

**Affiliations:** 1 Department of Critical Care Medicine, Universitair Ziekenhuis Brussel, Vrije Universiteit Brussel, Brussels, Belgium; 2 Department of Biostatistics, Vrije Universiteit Brussel, Brussels, Belgium; 3 Laboratory of Clinical Chemistry & Radioimmunology, Universitair Ziekenhuis Brussel, Vrije Universiteit Brussel, Brussels, Belgium; 4 Department of Intensive Care, Erasme Hospital, Université Libre de Bruxelles, Brussels, Belgium; Azienda Ospedaliero Universitaria Careggi, ITALY

## Abstract

The pathogenesis of delirium in critically ill patients is multifactorial. How hypotension and hypoxemia affect brain function and whether they can promote delirium remains unclear. A high cumulative positive fluid balance may also have a negative effect on brain function and promote delirium. We hypothesized that delirium would be more likely to develop in patients with low systemic arterial pressure, hypoxemia and a higher positive fluid balance, and investigated these associations in a prospective observational cohort study in patients with shock. After initial resuscitation, episodes of hypotension, defined as a mean arterial pressure (MAP) <65 mmHg or diastolic pressure <60 mmHg, and hypoxemia, defined as peripheral oxygen saturation (SpO_2_) <90% for more than one minute or any arterial oxygen concentration (PaO_2_) <90 mmHg, were recorded during the first 5 days of the ICU stay. Fluid balance was evaluated daily and the 5-day cumulative fluid balance recorded. Delirium was assessed using the Confusion Assessment Method for the ICU. A total of 252 patients were admitted with shock during the study period; 185 (73%) developed delirium. Patients who developed delirium also had more episodes of hypotension with a low MAP (p = 0.013) or diastolic pressure (p = 0.018) during the first five days of the ICU stay than those who did not. Patients with a higher cumulative fluid balance during the same period were also more likely to develop delirium (p = 0.01); there was no significant difference in the occurrence of hypoxemia between groups. Joint modeling, combining a linear-mixed model and an adjusted Cox survival model showed that low diastolic pressure (alpha effect = -0.058±0.0013, p = 0.043) and a positive cumulative fluid balance (alpha effect = 0.04±0.003, p = 0.021) were independently associated with delirium. In conclusion, low diastolic pressure and a cumulative positive fluid balance but not hypoxemia were independently associated with development of delirium in patients with shock.

## Introduction

The pathogenesis of delirium in critically ill patients is multifactorial. Administration of excessive sedative and analgesic agents is a known risk factor for delirium along with high disease severity [1, 2]. Systemic hypotension and hypoxemia have also been suggested as potential risk factors, but their role remains unclear because their definitions vary from study to study. Although maintaining a mean arterial pressure (MAP) ≥ 65 mmHg is usually suggested as sufficient to maintain adequate organ perfusion in clinical practice [3], whether reduction in MAP below this level affects brain function is unclear, particularly when it falls below the threshold for cerebral autoregulation, i.e., < 50 mmHg [3,4,5]. Moreover, is delirium associated more with a low MAP or a low diastolic arterial pressure, because diastolic pressure comprises almost two thirds of the total MAP and is important for perfusion of organs with low flow resistance, such as the brain [6]?

It has been shown that delirium is more frequent in patients with acute respiratory distress syndrome (ARDS) but it remains uncertain whether delirium is associate with hypoxemia per se [7].

Delirium has also been shown to occur more frequently after excessive fluid administration in post-surgical patients [8] and in patients with subarachnoid hemorrhage [9]. Indeed, although fluid administration is necessary for correction of hypotension in shock, excessive fluid administration can impair organ function and worsen outcomes [10, 11, 12]. Hence, a positive fluid balance may have negative effects on brain function and delirium, especially in patients with blood-brain-barrier (BBB) leakage (9), which allows greater passage of fluid into the brain. BBB leakage facilitates the passage of brain S100B protein into the serum, so elevated S100B levels have been used as a marker of brain injury or BBB leakage [13, 14, 15].

We therefore performed a prospective observational cohort study in patients with shock to investigate the development of delirium and its association with hypoxemia, fluid balance and hypotension. We hypothesized that delirium would be more likely to develop in patients with low systemic MAP and low diastolic pressure, hypoxemia and a higher positive fluid balance following shock resuscitation.

## Materials and methods

### Patients and definitions

The study was approved by the ethics committee of Universitair Ziekenhuis Brussel. Written informed consent was obtained from the patient or from the next of kin if the patient was incompetent. All patients admitted to the 24-bed multidisciplinary Department of Intensive Care with shock between September 2010 and April 2013 were considered for inclusion.

### Definitions

Shock was defined as systemic arterial hypotension with a MAP <65 mmHg associated with a blood lactate level >1.5 mmol/L and at least two of the following clinical signs: tachycardia, presence of clinical signs of tissue hypoperfusion (cold and cyanotic extremities, urine output <0.5 ml/kg body weight/hour). The etiology of shock was identified as cardiogenic, hypovolemic, and distributive/septic [16]. Septic shock was defined according to international consensus guidelines [17]. Cardiogenic shock was defined as shock associated with left ventricular dysfunction (ejection fraction <40%) and cardiac index <2.2 L/min/m^2^ following acute coronary syndrome or post-cardiotomy and demonstrated by echocardiography or right heart catheterization [18]. Hemorrhagic shock was defined as shock due to acute marked blood loss (gastrointestinal bleeding, post-partum or postsurgical bleeding, aortic or splenic rupture).

Hypotension was defined as a MAP <65 mmHg. MAP < 50 mmHg was considered as the lower threshold for presence of cerebral autoregulation [19]. Diastolic hypotension was defined as a diastolic pressure <60 mmHg [20]. ARDS and pulmonary edema were defined using standard criteria [21]. Hypoxemia was considered as a PaO_2_ <90 mmHg or any episode of SpO_2_ <90% lasting for more than one minute after excluding errors or technical faults. A patient was considered to have acute renal failure if their Sequential Organ Failure Assessment (SOFA) renal subscore was ≥2 (serum creatinine ≥2–3.4mg/dl) or they required continuous renal replacement therapy [22]. Nosocomial infection was defined as a documented infection occurring more than 48–72 hours after ICU admission or up to three days after ICU discharge, and for which initiation of or a change in antibiotherapy was needed [23].

### Exclusion criteria

Pregnant patients, patients <18 years old, patients with delirium at ICU admission and patients included in another study were excluded. To limit variation in exposure to risk factors for delirium, patients who were discharged or died after <5 days in the ICU were excluded. Patients considered to have poor short-term outcomes because of lack of an effective therapeutic option, e.g., those with cardiogenic shock following inoperable valvular disease or terminal coronary disease, were not included. Patients with obstructive shock following pulmonary embolus were also not included. Patients who developed delirium and had a metabolic disorder that can be associated with delirium (severe untreated hypothyroidism, liver cirrhosis with hyperammoniemia and chronic hemodialysis with hyperuremia, acute alcohol or drug intoxication or withdrawal) were excluded because it was difficult to determine the etiology of the delirium. A previous or current history of alcohol abuse was not per se a study exclusion criterion. Patients who developed delirium and had a history of a primary neurological disorder that can be associated with delirium (cerebral trauma, stroke, cerebral hemorrhage, meningitis, post-neurosurgery, post-cardiorespiratory arrest, severe psychiatric disorders, dementia) were excluded, again because it would have been difficult to determine the etiology of the delirium. However, a history of these conditions without residual cognitive dysfunction or neurological sequelae was not per se exclusion criterion.

Patients with a likely extracerebral source of serum S100B elevation (e.g., severe disabling neuromuscular disorders, severe burns, advanced malignancy, polytrauma, and chronic renal failure) were excluded [24].

### Monitoring and measurements

Severity of illness was assessed on admission using the Acute Physiology and Chronic Health Evaluation (APACHE III) score. The degree of organ dysfunction was assessed daily using the SOFA score for the first five days after ICU admission.

After initial resuscitation, arterial pressure was monitored continuously using a radial or femoral arterial catheter and the highest/lowest values of diastolic pressure and MAP were recorded hourly. Heart rate and central venous pressure (CVP) were also recorded hourly. Peripheral oxygen saturation (SpO_2_) was monitored continuously by pulse oximetry. Arterial blood gases (pH, PaO_2_, PaCO_2_) were checked at least three times daily.

Fluid balance was assessed daily for 5 days. Fluid output was calculated as the sum of the volumes of urine output, ultrafiltration, all drain fluid volumes, and estimated gastrointestinal losses; insensitive losses were not included. Fluid input was calculated as the sum of all intravenous and oral fluids [12]. Daily fluid balance was calculated as the difference between the total daily fluid output and input. Cumulative fluid balance was the total fluid balance over the 5 days after ICU admission.

### Shock management

The initial management of shock in our Department follows the VIP rule: ventilate (oxygen administration or mechanical ventilation), infuse (fluid resuscitation) and pump (vasoactive agent administration) [16]. Patients were treated to achieve an MAP ≥65 mm Hg with adequate oxygenation, improved peripheral perfusion, and blood lactate <2 mmol/L, using fluid administration (colloids and crystalloids) combined with norepinephrine (up to 2–3 μg/kg/min). Dobutamine (≤10 μg/kg/min) was used to increase cardiac output when indicated. Epinephrine or terlipressin was added as a second-line therapeutic agent in case of refractory shock not responding to norepinephrine and dobutamine. Blood and plasma transfusions were administered when necessary, especially in patients with hemorrhagic shock [25].

Patients with septic shock were managed according to international consensus guidelines [26]. Patients with cardiogenic shock were managed according to the American Heart Association and European Society of Cardiology guidelines for the management of patients with ST-elevation myocardial infarction [27, 28]. When indicated, emergency reperfusion was achieved by percutaneous coronary angioplasty and stenting or by by-pass surgery in acute coronary syndrome. Patients with hemorrhagic shock were managed according to the European guidelines for advanced bleeding in trauma [25] and the primary source of hemorrhage was controlled by surgery, angiographic embolization or endoscopy. Mechanical support, including extracorporeal membrane oxygenation (ECMO) and intra-aortic balloon pump, was used when indicated.

Echocardiography was performed in all patients to aid diagnosis or to evaluate cardiac function and predict fluid responsiveness. Right heart catheterization was performed when indicated to monitor these parameters after echocardiographic examination [29]. In all patients receiving mechanical ventilation, a tidal volume of 6–7 mL/kg ideal body weight and a plateau pressure <30 cmH_2_O was targeted. When required, sedation was achieved with propofol or midazolam and analgesia with morphine, remifentanil or fentanyl.

### Delirium assessment

In non-sedated patients, the Glasgow Coma Scale (GCS) was evaluated twice daily from admission until ICU discharge by the nurse in charge. Coma was considered when a GCS <8 was observed in the absence of any sedation. In non-comatose patients, the Richmond Agitation Sedation Scale (RASS) score and the Confusion Assessment Method for the ICU (CAM-ICU) were assessed twice daily until ICU discharge. Delirium was diagnosed when, in a patient with a RASS score >-3 to exclude any residual effect of sedation and analgesia, the CAM-ICU was positive for at least two consecutive days. Patients were considered to have hyperactive delirium when they had a positive CAM-ICU combined with a RASS score of 1 to 5, and hypoactive delirium if the RASS score was -2 or -1. Mixed delirium was considered when the patient had both delirium subtypes [30, 31]. Brain CT was performed to exclude structural brain injury in patients with a GCS <10 or who had hypoactive or mixed delirium with a persistently fluctuating RASS score between -3 and -4 in the absence of any sedation.

### Serum S100B protein measurement

S100B protein (Immunoradiometric assay, Roche Diagnostics GmbH, Germany) was measured 12–24 hours after ICU admission, and then daily over the subsequent two days [24]. S100B was also measured in the cerebrospinal fluid in patients who underwent a lumbar puncture to confirm its brain origin. Normal values of serum S100B in our laboratory are ≤ 0.105 μg/L.

### Statistical analysis

Statistical analysis was performed using SPSS 23 (SPSS, Chicago, IL) and the package JM of R software (R foundation for statistical computing, New Zealand). Categorical variables were compared using a chi-square test. Student’s T-tests, linear mixed models (with 'patient' as a random effect) adjusted for covariates (age, sex, history of arterial hypertension) or repeated measures analysis of variance with Bonferroni post-hoc testing were used when appropriate for comparisons between groups. A non-parametric Wilcoxon sum rank-test was used for skewed variables after logarithmic transformations (S100B) for comparisons between groups.

To determine the risk factors associated with the development of delirium and taking into account the time-dependent covariates and correlations between repeated measurements of a covariate in a same subject, joint modeling was performed using the JM package in the R statistic software (R foundation for statistical computing, New Zealand) [32]. The joint modeling combined a linear-mixed model and a survival Cox proportional hazards model, and adjusted for covariates commonly reported to be associated with the development of delirium (sex, age, admission SOFA score, duration of sedation, a history of excessive alcohol intake, and a history of neurological disorder) [2, 8, 33]. This method evaluated the association alpha effect of low diastolic arterial pressure, MAP, cumulative fluid balance and low PaO_2_ with the development of delirium. The response covariate was the length of time (in days) from ICU admission to the development of delirium. Statistical significance was considered if the two sided p-value was <0.05.

## Results

**Fig 1:**
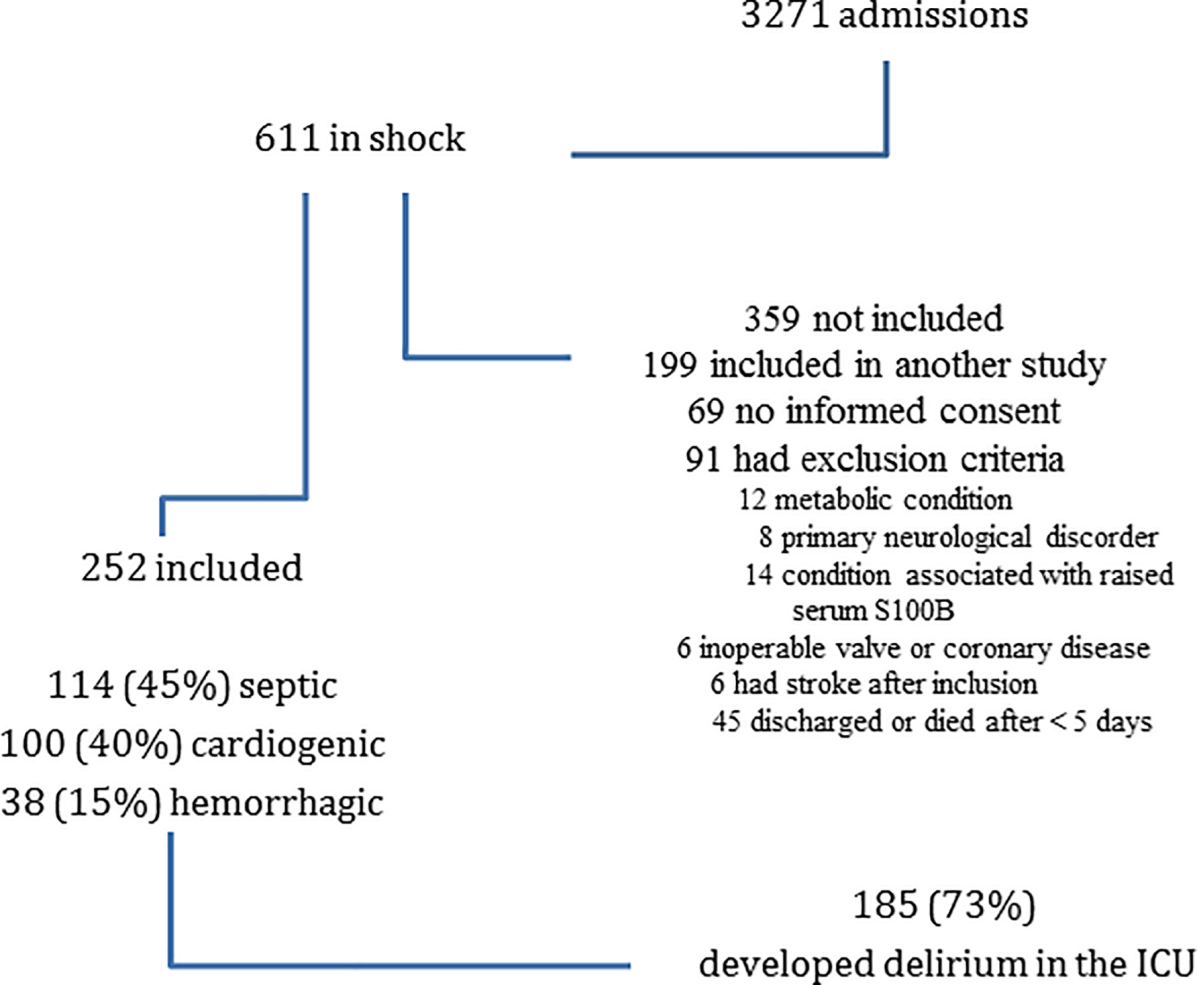
Patient inclusion and outcomes.

A total of 3271 patients were admitted to the ICU during the study period, of whom 611 had shock; 359 of these patients were not included (91 met exclusion criteria, 69 had no informed consent, and 199 were included in another study), leaving 252 patients for analysis (114 (45%) with shock of septic origin, 100 (40%) cardiogenic and 38 (15%) hemorrhagic) (Fig 1). The characteristics of the included patients on admission are shown in Table 1. No patient had aortic insufficiency on echocardiography.

**Table 1:** Characteristics of patients at ICU admission according to whether or not they developed delirium.

Characteristic	All patients n = 252	No-delirium n = 67 (27%)	Delirium n = 185 (73%)
**Male, n (%)**	168 (67)	39 (58)	129 (70)
**Age, years (mean ± SD)**	68 ± 14	62 ± 15	70 ± 12
**Body mass index (mean ± SD)**	27 ± 10	26 ± 6	28 ± 11
**Septic shock, n (%)**	114 (45)	28 (42)	86 (46)
**Cardiogenic shock,** **n (%)**	100 (40)	31 (46)	69 (37)
**Hemorrhagic shock, n (%)**	38 (15)	8 (12)	30 (16)
***Comorbidities***			
**Diabetes, n (%)**	59 (23)	11 (16)	48 (26)
**Arterial hypertension, n (%)**	145 (58)	35 (52)	110 (60)
**Ischemic and non-ischemic** **cardiopathy, n (%)**	119 (47)	33 (49)	86 (47)
**Dyslipidemia, n (%)**	97 (39)	27 (40)	70 (38)
**History of atrial fibrillation, n (%)**	41 (16)	13 (19)	28 (15)
**History of previous sepsis, n (%)**	55 (22)	16 (24)	39 (21)
**History of neurological disorder, n (%)**	65 (26)	11 (16)	54 (29)
**Chronic renal failure, n (%)**	50 (20)	9 (13)	41 (22)
**Smoking, n (%)**	47 (19)	19 (28)	48 (26)
**History of excessive alcohol intake, n (%)**	25 (10)	2 (3)	23 (12)
***Medications before admission***			
**Corticosteroids, n (%)**	135 (54)	33 (49)	102 (55)
**Angiotensin-converting-enzyme inhibitor, n (%)**	113 (45)	28 (42)	85 (46)
**Beta-blocker or** **calcium antagonist, n (%)**	124 (49)	29 (43)	95 (52)
**Benzodiazepines, n (%)**	67 (27)	20 (30)	47 (25)
**Statins, n (%)**	97 (39)	27 (40)	70 (38)
***Variables at ICU admission (mean ± SD)***			
**Glasgow Coma Scale (GCS)**	13 ± 2	14 ± 3	13 ± 4
**APACHE III scores**	78 ± 31	73 ± 32	80 ± 31
**In-hospital length of stay before ICU admission, days**	4 ± 7	4 ± 8	4 ± 6
**SOFA score without GCS**	7 ± 3	6 ± 3	7 ± 3
**Cardiovascular SOFA**	3 ± 2	2 ± 2	3 ± 1
**Renal SOFA**	1 ± 1	1 ± 1	1 ± 1
**Respiratory SOFA**	3 ± 1	2 ± 1	3 ± 1
***Variables in the first 24 H after ICU admission (mean ± SD)***			
**Lowest pH**	7.29 ± 0.10	7.29 ± 0.11	7.30 ± 0.10
**Highest CO_2_, mm Hg**	47 ± 25	48 ± 12	48 ± 28
**Lowest CO_2_, mm Hg**	36 ± 7	35 ± 7	36 ± 7
**Lowest PaO_2_, mm Hg**	96 ± 32	92 ± 30	98 ± 33
**PaO_2_/FiO_2_ ratio**	176 ± 99	175 ± 100	175 ± 94
**C-reactive protein, mg/dl**	126 ± 131	124 ± 128	127 ± 132
**Lactate, mmol/L**	3.2 ± 3.2	3.2 ± 2.8	3.2 ± 3.4
**Hemoglobin, g/L**	10 ± 3	10 ± 2	10 ± 3
**Serum creatinine, mg/dl**	1.5 ± 1.1	1.6 ± 1.4	1.5 ± 0.85
**Lowest systolic pressure,** **mm Hg**	87 ± 20	86 ± 19	87 ± 20
**Lowest diastolic pressure, mm Hg**	47 ± 13	48 ± 14	47 ± 12
**Lowest mean arterial pressure, mm Hg**	61 ± 14	61 ± 12	61 ± 14
**Highest heart rate, beats/min**	109 ± 24	108 ± 22	109 ± 25
**Lowest central venous pressure, mm Hg**	12 ± 5	12 ± 4	12 ± 5
**Highest central venous pressure, mm Hg**	18 ± 5	17 ± 5	18 ± 5
**Fluid balance, ml**	2422 ± 2445	2535 ± 2311	2382 ± 2496

One hundred and eighty-five of these patients (73%) developed delirium during the ICU stay: 103 patients (56%) with hypoactive delirium, 59 (32%) with hyperactive and 23 (12%) with mixed delirium. The median duration of the delirium was 4 (2–8) days. Patients who developed delirium were older (70±12 vs. 62±15 years old, p = 0.001), had lower GCS (13±4 vs. 14±3, p = 0.044), and higher SOFA (7±3 vs. 6±3, p = 0.01) scores on admission, and were more likely to have a history of neurological disorder (54 (29%) vs. 11 (16%), p = 0.041) or of excessive alcohol intake (23 (12%) vs. 2 (3%), p = 0.027) than patients who did not develop delirium. Ten of the patients who developed delirium had a lumbar puncture to exclude meningoencephalitis.

### Impact of hypoxemia, hypotension, and fluid balance on the development of delirium

#### Hypoxemia

There were no significant differences in the numbers of episodes of SpO_2_ <90% or in the highest/lowest values of blood pH, PaO_2_ or PaCO_2_ over the first five days of observation in patients who developed delirium and those who did not.

#### Hypotension

**Fig 2:**
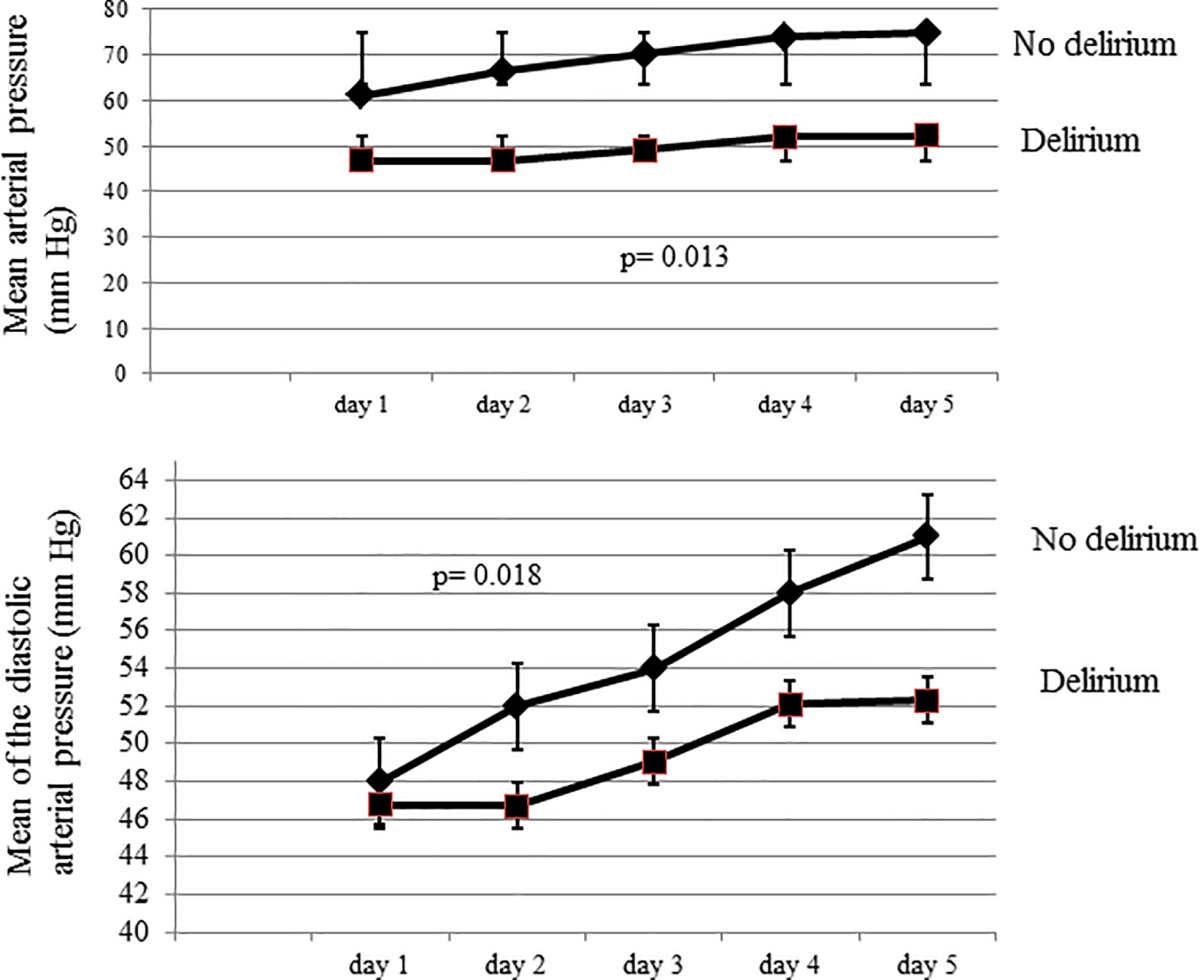
Mean arterial pressure (top panel) and mean diastolic pressure (lower panel) over the first 5 days of the ICU stay in patients who developed delirium and those who did not.

**Fig 3:**
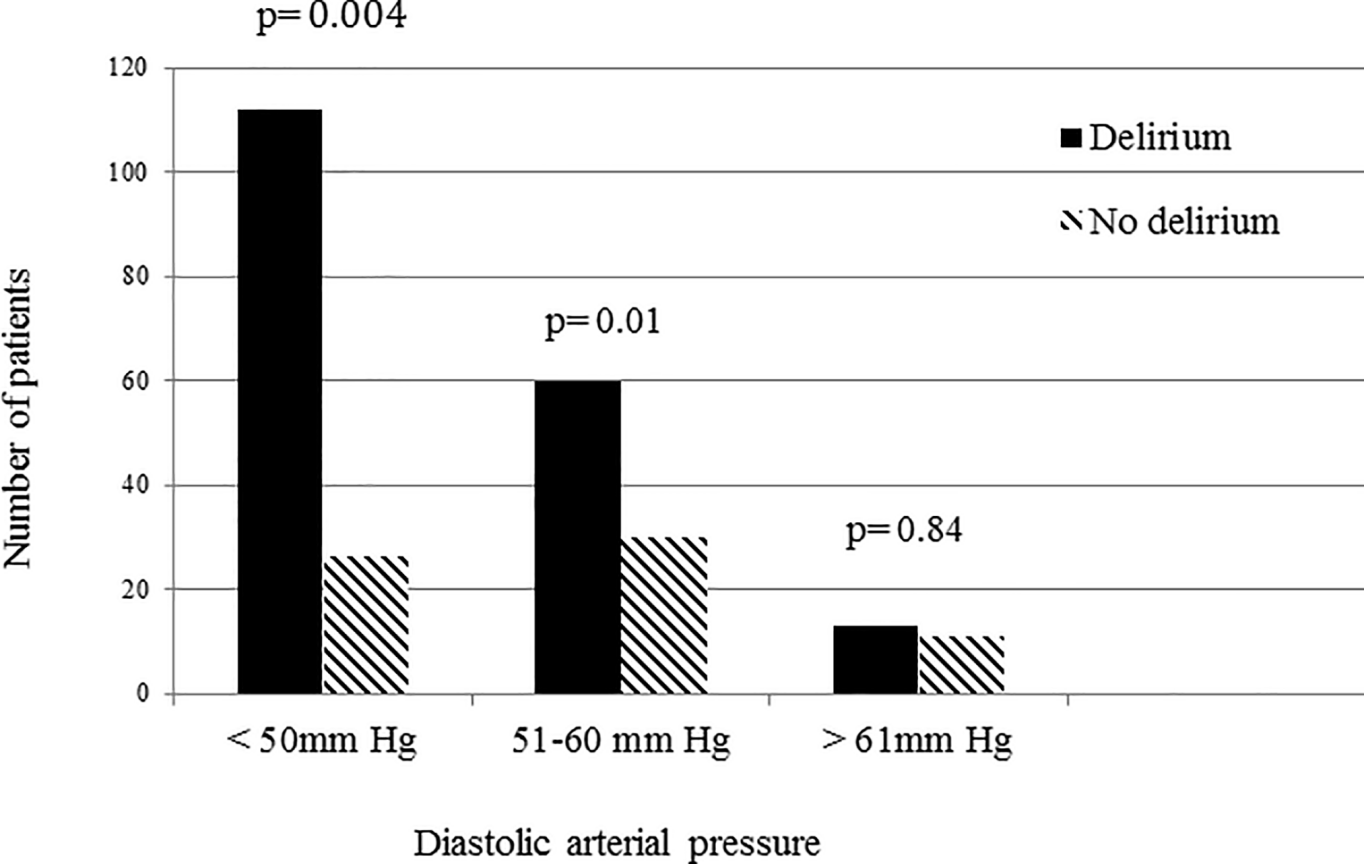
The incidence of delirium according to the presence of diastolic hypotension at any point during the first 5 days of the ICU stay.

**Fig 4:**
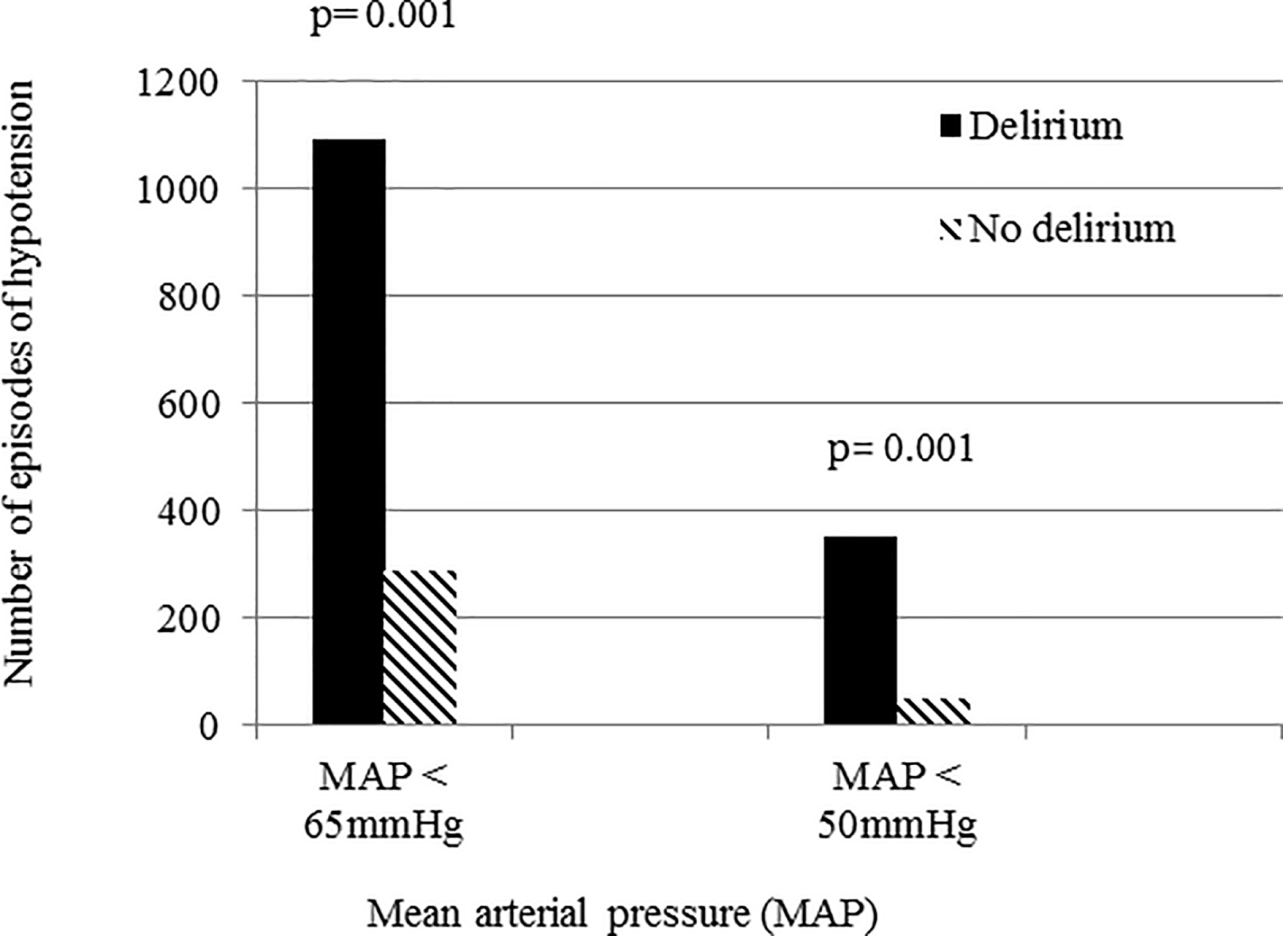
Number of episodes of hypotension in patients who developed delirium and those who did not.

Patients who developed delirium had lower average MAP (p = 0.013) and diastolic pressures (p = 0.018) over the 5-day period than those who did not (Fig 2). In patients with at least one episode of diastolic pressure <50 mmHg during the study period, 81% developed delirium compared to 7% in those with no diastolic pressure < 50 mmHg (p = 0.004) (Fig 3). Patients who developed delirium also had more episodes of hypotension with MAP <65 mmHg (1094 vs. 233 episodes, p = 0.001) and MAP <50 mmHg (351 vs. 53 episodes, p = 0.001) than those who did not develop delirium(Fig 4).

#### Fluid balance

**Fig 5:**
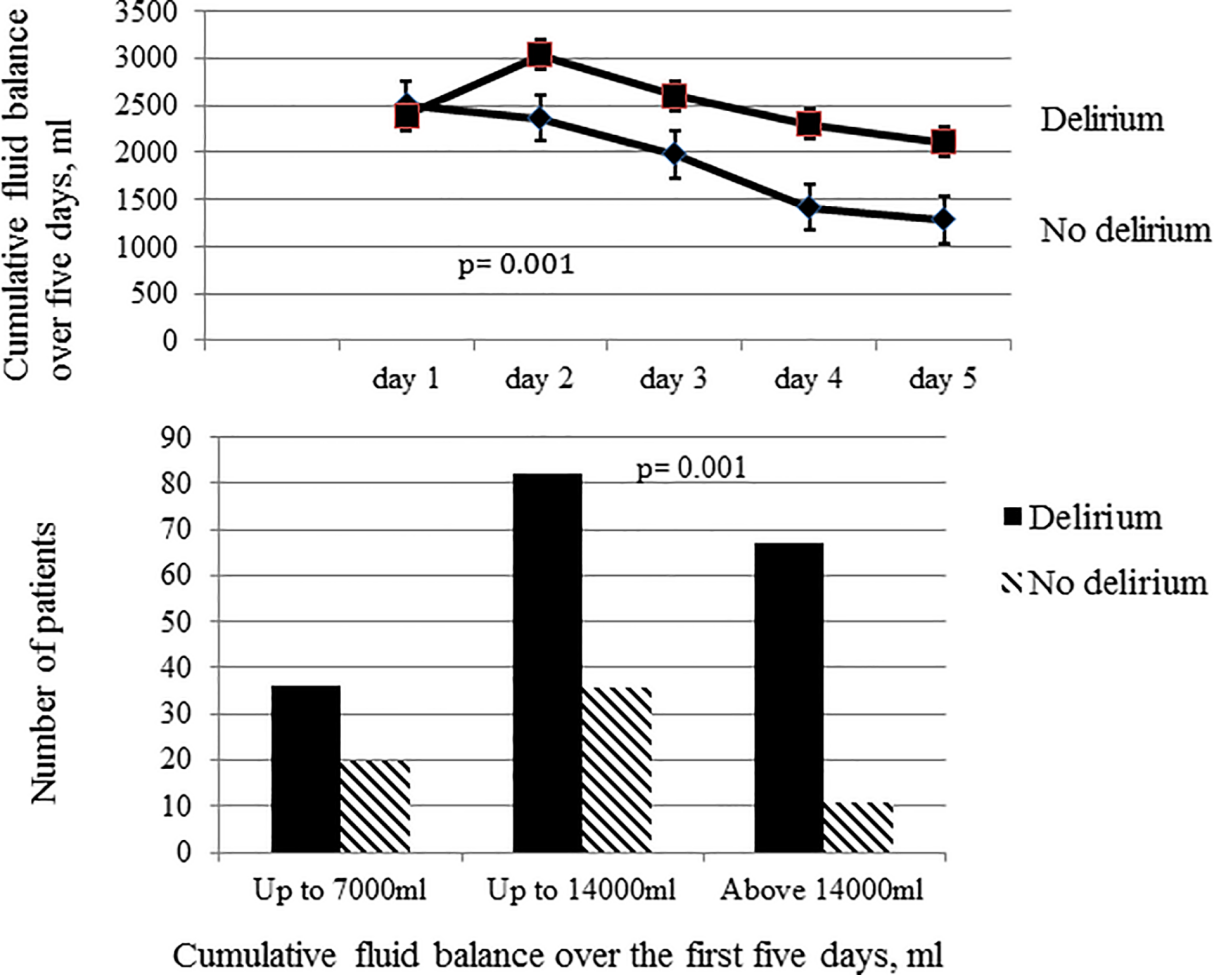
Cumulative fluid balance over the first five days in patients who developed delirium and those who did not (top panel). Incidence of delirium according to cumulative fluid balance over the first five days of ICU stay (lower panel).

For the entire cohort, the total cumulative fluid balance over the five days was 11755±7024 ml (160±100 ml/kg). The mean cumulative fluid balance over the 5 days was greater in patients who developed delirium than in those who did not: 12716±7342 ml vs. 9177±5290 ml (171±104 vs. 128±80 ml/kg; both p = 0.001) (Fig 5). There were no differences in CVP values between the two groups of patients during the study period. The cut-off fluid balance for the development of delirium on the ROC curve was 6994 ml (86 ml/kg) (81% sensitivity, 70% specificity, area under the curve (AUC) 0.73 [95% CI: 0.65, 0.78], p = 0.001). The incidence of delirium was twice as high in patients with a positive fluid balance >7000 ml than in those with lower fluid balances (Fig 5).

### ICU evolution and mortality

Two hundred and forty-two patients required mechanical ventilation (96%) and 94 patients (37%) died during the ICU stay. Durations of sedation and mechanical ventilation were longer in patients who developed delirium than in those who did not (Table 2). Patients who developed delirium received lower doses of morphine over the 5 days but higher doses of dobutamine and norepinephrine. There were no differences in the occurrence of acute kidney injury or ARDS in the two groups (data not shown). Patients who developed delirium had a higher incidence of nosocomial infection (116 (63%) vs. 67 (27%), p = 001), longer length of ICU stay (median (interquartile range): 15 (9, 27) vs. 9 (5, 21) days, p = 0.002), and higher ICU (84 (45% vs. 10 (15%), p = 0.001) and in-hospital (16 (16%) vs. 2 (4%), p = 0.019) mortality.

**Table 2:** Duration of sedation and doses of sedative and analgesic agents according to development of delirium.

	All patients n = 252	No-delirium n = 67 (27%)	Delirium n = 185 (73%)	p-value
*ICU parameters (median [interquartile range])*				
Duration of mechanical ventilation, days	7 (4, 18)	5 (2, 9)	8 (4, 22)	**0.001**
Duration of sedation, days	6 (3, 12)	4 (2, 7)	7 (4, 13)	**0.001**
Maximum dose of midazolam in the first five days, mg/hour	3.4 (0, 5.4)	2 (0, 5.8)	3.6 (0.8, 5.4)	0.084
Maximum dose of propofol in the first five days, mg/hour	1 (1, 3.2)	0.8 (1, 3.1)	1 (1, 3.2)	0.866
Maximum dose of fentanyl in the first five days, mg/hour	1.6 (0, 2.4)	1.2 (0, 2.4)	1.6 (0, 2.4)	0.516
Maximum dose of morphine in the first five days, mg/hour	1 (0.5, 1.5)	1 (1, 1.5)	0.5 (0. 6,1)	**0.044**
Maximum dose of remifentanil in the first five days, mg/hour	0.1 (0, 0.2)	0.07 (0, 0.2)	0.1 (0, 0.2)	0.081
Maximum dose of dobutamine in the first five days, μg/kg/min	2.2 (0, 5.3)	1.4 (0, 8)	2.8 (0, 5.8)	**0.038**
Maximum dose of norepinephrine in the first five days, μg/kg/min	0.1 (0, 0.21)	0.07 (0, 0.15)	0.1 (0, 0.22)	**0.01**

Patients who developed delirium had higher serum S100B levels during the first three days in the ICU than those who did not (p = 0.01). Ten of the patients who developed delirium had a lumbar puncture to exclude meningoencephalitis. The median (IQ) value of CSF S100B in these 10 patients was 0.21 (0.16, 0.35) μg/L; their serum S100B level measured at the same time was >0.105 μg/L.

Among the 185 patients who developed delirium, 84 (45%) had a brain CT scan during the ICU stay; the scan was abnormal in 47 of these patients (56%) with a small ischemic or hemorrhagic lesion in 19 and 3 patients, respectively, and cortical sub-cortical atrophy in 25 patients.

### Risk factors associated with development of delirium

Using joint modeling, a diastolic pressure <60 mmHg, a positive 5-day cumulative fluid balance and older age were significantly associated with the development of delirium (Table 3). MAP <65 mmHg and PaO_2_ <90 mmHg were not significantly associated with the development of delirium. The correlation coefficient between the factors of ‘diastolic pressure’ and ‘MAP’ was 0.70, and between the factor of ‘fluid balance’ and both the diastolic pressure and the MAP was <0.5.

**Table 3:** Results of joint modeling to determine the risk factors associated with the development of delirium.

Variable	Association alpha effect	Standard error	p-value
Diastolic arterial pressure < 60 mmHg	-0.058	0.0013	**0.043**
Mean arterial pressure < 65 mmHg	-0.018	0.0009	0.111
Positive cumulative fluid balance at day five	0.04	0.003	**0.021**
PaO_2_ < 90 mmHg	-0.005	0.0004	0.212
Age (per year)	0.233	0.021	**0.011**

## Discussion

In this cohort study, 73% patients with shock developed delirium during the ICU stay. Low diastolic pressure and a cumulative positive fluid balance, but not hypoxemia, were independently associated with development of delirium in patients with shock. Older age was also independently associated with the development of delirium.

The role of hypotension in the development of delirium, if any, remains unclear, because the definition of hypotension and the study populations (e.g., neurosurgical vs. septic) vary [33–36]. In a dog model of hemorrhagic shock, reduction in MAP to ≤40 mmHg induced brain dysfunction [37], but it is difficult to translate this to the clinical situation as prompt shock resuscitation would be undertaken in patients before this degree of hypotension develops. Patients with shock who developed delirium required higher doses of norepinephrine and dobutamine. These patients also had more episodes of hypotension and persistently lower MAP values during the 5 days after ICU admission than patients without delirium. Hirsch et al. also showed that recurrent hypotension, but not the degree of MAP reduction during the intraoperative period, was associated with the development of delirium [38]. Moreover, it has been shown that recurrent hypotension can induce hippocampal lesions in experimental rat studies [39]. In contrast to the findings of Hirsch et al., we found that the degree of MAP reduction was also involved in the development of delirium. Delirium occurred more frequently in patients with MAP below the 50 mmHg threshold of cerebral autoregulation than in those with MAP <65 mmHg. Cerebral autoregulation enables a stable cerebral blood flow to be maintained for MAP values between 50–150 mmHg. Thus, when the MAP decreases <50 mmHg brain hypoperfusion and ischemia may be induced. Importantly, although the lower limit of autoregulation may be higher in hypertensive patients due to the right shift of the autoregulation curve [20]. In our study, the occurrence of delirium was similar in patients with and without a history of chronic arterial hypertension.

Although diastolic pressure is a major component of MAP, our results show that a low diastolic pressure was independently associated with the development of delirium but low MAP was not. Diastolic pressure is important for perfusion of organs with low flow resistance, such as the brain; low diastolic values reflect higher degrees of arterial stiffness associated with cerebrovascular atherosclerosis, regional cerebral blood volume and microcirculation disturbance [40]. Hence, low diastolic pressure in shock may compromise regional cerebral perfusion and contribute to delirium, especially in elderly patients [41]. Increased systolic pressure may result in increased MAP but not in increased diastolic pressure. It has been shown that a diastolic pressure <60 mmHg is associated with higher mortality and can aggravate the progression of brain atrophy in patients with cardiovascular disease [42–44], and increase cognitive decline, the risk of dementia and mortality rates in hypertensive elderly patients [45,46]. A low diastolic pressure has also been associated with the development of acute kidney injury (AKI) in ICU patients [47] and further worsened kidney function in patients with chronic kidney disease [48].

Excessive fluid administration for shock resuscitation resulting in a positive fluid balance >7000 ml by day 5 was independently associated with development of delirium. Others have shown that a positive fluid balance aggravated delirium in post-surgical patients and worsened outcomes in patients with subarachnoid hemorrhage [8, 9]. Fluid accumulation with brain vasogenic edema formation, but not venous congestion, as has been reported in patients with AKI [47], could explain the increased risk of delirium in our patients because no differences in CVP values were found in patients with and without delirium. Using S100B as a marker of BBB leakage, we found that patients who developed delirium more likely had BBB leakage as reflected by higher serum S100B levels [15]. This leakage enables greater passage of fluids and neurotoxic substances into the brain, and excessive fluid administration could, therefore, promote brain edema formation and aggravate hypoxic ischemia [49]. In septic shock, Sharshar et al. showed that brain edema occurred with increased white matter water content on magnetic resonance imaging (MRI) [50]. The same authors also showed, in post-mortem examination of sepsis non-survivors, that shock induced brain hypoxic-ischemic lesions with BBB leakage [51]. Brain perivessel edema with increased brain water content has also been shown in various experimental septic or hypovolemic shock models [52, 53]. In the present study, no brain edema was seen on CT scans in any patient with delirium, although this imaging technique is less sensitive than MRI for detecting edema.

Delirium has been associated with hypoxemia and ARDS in some [7], but not all [54], studies. In our patients, hypoxemia was not independently associated with the development of delirium. The Cerebral Oxygenation and Neurological Outcomes Following Critical Illness (CONFOCAL) researchers reported that low brain tissue oxygenation, measured using near-infrared spectroscopy (NIRS), may be associated with the development of delirium, but this technique is not widely available and brain tissue oxygenation was not measured in our patients [55].

Our study has several other limitations. First, this was a single-center study so that the results may not be generalizable to other ICUs. Second, the impact of pre-ICU admission hypotension and hypoxemia cannot be determined. The duration of hypotension was also not reported. Cerebral blood flow or perfusion pressure were not measured to investigate the direct impact of hypotension on brain dysfunction although increased serum S100B values can reflect BBB leakage and/or evolving brain injury in critically ill patients [56]. S100B can be released by organs other than the brain, but patients with clinical conditions known to be implicated in extracranial release of this biomarker (bone fracture, burns with adipose tissue necrosis) were excluded [19, 57]. Moreover, concomitant elevation of S100B in the serum and in the cerebrospinal fluid (when measured) in patients who developed delirium argue against an extracerebral source of this biomarker. Finally, the statistical association we identified between hypotension or fluid balance and delirium does not necessarily imply causality.

## Conclusions

A low diastolic pressure (< 60 mmHg) and a positive 5-day cumulative fluid balance, but not hypoxemia, were independently associated with the development of delirium in patients with shock.

## Supporting information

S1 File Clinical data of hypotension and delirium in shock in excel file available. (XLSX)
